# Mitomycin C induces bystander killing in homogeneous and heterogeneous hepatoma cellular models

**DOI:** 10.1186/1476-4598-8-87

**Published:** 2009-10-21

**Authors:** Ratna Kumari, Aanchal Sharma, Amrendra Kumar Ajay, Manoj Kumar Bhat

**Affiliations:** 1 National Centre for Cell Science, Ganeshkhind, Pune- 411 007, India

## Abstract

**Background:**

Hepatocellular carcinoma (HCC) is one of the most common cancers worldwide that is particularly refractory to chemotherapy. Several studies have proposed combination chemotherapy regimen for HCC treatment. However, these therapies are not effective in regressing tumor and prolonging survival of patient's suffering from HCC. Therefore, the development of more effective therapeutic tools and new strategies for the treatment of HCC are urgently needed. Over the last decade much attention has been focused on "bystander effect" as a possible therapeutic strategy for the treatment of certain human tumors. Interest in this therapeutic approach originated from numerous reports describing the radiation induced bystander effect. However, the knowledge about chemotherapy induced bystander effect is still limited. Hence, chemotherapy induced bystander phenomenon in hepatoma cells was explored by utilizing Mitomycin C (MMC).

**Results:**

MMC induced bystander killing was observed only in hepatoma cells and it did not occur in cervical cancer cells. MMC induced bystander killing was transferable via medium. It occurred in co-cultured cells indicating the involvement of secreted as well as membrane bound factors. FasL and TRAIL were detected in the conditioned medium from treated cells. In medium transfer experiment, pre-treatment with EDTA (a broad range protease inhibitor) diminished MMC induced bystander killing. Following drug exposure, expression of Fas and TRAIL receptors increased and treatment with neutralizing antibodies against FasL and TRAIL inhibited bystander killing.

**Conclusion:**

Our results highlight the therapeutic importance of MMC in the treatment of HCC and implicate role of membrane bound and secreted forms of FasL and TRAIL in MMC induced bystander killing.

## Background

For solid tumors chemotherapy is characteristically the choice of treatment other than surgical resection. However, there are inherent limitations associated with chemotherapy of solid tumors. These include toxicity to normal cells, development of resistant cells within a tumor mass and inadequate delivery of cytotoxic drugs due to inaccessibility to certain cells within the same tumor mass. Moreover, the effectiveness of a chemotherapeutic drug is a combined characteristic of the drug, tumor and patient. The universal adoption of cancer chemotherapy is limited by treatment complexities which make it mandatory to explore drugs that, instead of assuring delivery to 100% of tumor cells, utilize the ability of exposed cells to kill neighboring unexposed tumor cells. Therefore, the concept of "Bystander Effect" comes into the picture. The name was initially borrowed from the field of gene therapy, where it usually referred to the killing of several types of tumor cells by targeting only one type of cell within a mixed population [1]. Radiation induced bystander effect has been extensively studied [2-4]. The available data concerning this effect falls into two categories: (i) confluent cultures where physical contacts between irradiated and non-irradiated cells are made and where intercellular communication is essential for the process and (ii) sparsely populated cultures where the bystander effect may be mediated by damage signals released into the culture medium by irradiated cells' [4-7].

Hepatocellular carcinoma (HCC) is one of the most common solid tumors that are particularly refractory to the available chemotherapeutic drugs administered either alone or in combination [8]. During the last few decades many drugs have become available for treatment of a wide variety of neoplastic diseases. Mitomycin C (MMC) is one such drug used for treating solid tumors including hepatocellular carcinoma and cervical cancer [9-12]. It is already known that chemotherapeutic DNA-alkylating agents can induce *in vivo *and *in vitro *paracrine factors that cause growth inhibition. In most cases there is production of soluble factors which have a distal effect on untreated cells [13]. Moreover, single acute exposure of MMC has been implicated in the changes in genomic stability of exposed cells that signals the unexposed bystander cells to respond similarly [14]. Various molecules that are involved in the genesis of bystander effect remain unidentified. There are reports demonstrating involvement of reactive oxygen species, nitric oxide and cytokines such as TGF β that promote bystander cell killing in sparsely populated cell cultures [15,16].

HCCs are heterogeneous tumors. They commonly emerge on the histological background of chronic liver disease. Heterogeneity of a tumor occurs due to clonal variations within a tumor mass during its development. However, in spite of heterogeneity, there are certain common molecular alterations in all sub-clones within a tumor that correlates with the monoclonal origin or homogeneity of a tumor. These molecular alterations and signaling pathways play a crucial role in development of HCC and also can be explored to develop an effective therapeutic tool for treatment of HCC [17].

Our study reveals the mechanism of bystander killing by MMC in hepatoma cells. We have earlier reported that 5-fluorouracil induces bystander killing in mixed co-cultures of breast cancer cells, a heterogeneous cell model [18]. In the present work we addressed the ability of MMC to specifically induce bystander killing of hepatoma cells in both homogeneous and heterogeneous cell models. We investigated the involvement of death ligands in the bystander killing of hepatoma cells. Interestingly, MMC induced bystander killing was transferable through medium and it occurred also in co-cultured cells, implicating the involvement of soluble as well as membrane bound death effector molecules.

## Methods

### Cell lines and reagents

Hepatoma cell lines HepG2, Hep3B and cervical cancer cell lines HeLa, SiHa were obtained from American Type Culture Collection (Manassas, VA, USA) and maintained in our in-house National Cell Repository. Cells were routinely cultured in minimum essential medium (MEM) supplemented with 10% heat inactivated fetal bovine serum (Sigma, MO, USA), penicillin (100 U/ml) and streptomycin (100 μg/ml) (Invitrogen Corporation, CA, USA) at 37°C with 5% CO_2_. Chemotherapeutic drug Mitomycin C (MMC), purchased from Sigma was dissolved in methanol to make a stock solution of 5 mM. Methylethiazole tetrazolium (MTT) (USB, OH, USA) was reconstituted in DMEM without phenol red to make 1 mg/ml working solution. Antibodies against Fas, FasL, TRAIL, DR4, caspase-3, BID, PARP, β-actin, recombinant human FasL, HRP conjugated and FITC-conjugated secondary antibodies were purchased from Santa Cruz Biotechnology (CA, USA). Neutralizing antibodies against FasL and TRAIL (BD Biosciences, CA, USA) were reconstituted in sterile PBS to make 1 mg/ml solution.

### Development of EGFP expressing cell lines

The cell lines, expressing enhanced green fluorescent protein (EGFP) were established by transfecting HepG2, HeLa and SiHa cells with pEGFPN1 plasmid (Clontech, CA, USA) by Lipofectamine 2000 (Invitrogen Corporation, CA, USA) as described in the manufacturer's protocol and selected on G418 (USB, OH, USA) 800 μg/ml (for HepG2) and 400 μg/ml (for HeLa and SiHa cells). These cells were subsequently maintained in medium containing G418 (100 μg/ml).

### Cell growth and cytotoxicity assay for HepG2 and HepG2-EGFP cell lines

Cells were seeded in a 96 well plate and the growth kinetics was performed as described previously [19]. To assay cytotoxicity of drug, cells seeded in a 96 well plate were treated with indicated concentration of MMC for 48 h and then cultured for additional 24 h in drug free medium. Subsequently, MTT assay was performed as described previously [20].

### Bystander cytotoxicity

*In vitro *co-culture experiments were designed to evaluate whether the cytotoxicity induced by MMC in HepG2, Hep3B, HeLa and SiHa cells (MMC treated cells were termed as "Effector Cells") causes death in co-plated bystander HepG2-EGFP, HeLa-EGFP and SiHa-EGFP cells (EGFP expressing bystander cells which were not treated with MMC were termed as "Target Cells"). Death of target cells was evaluated by quantification of total live GFP fluorescence by exciting at 485 nm and measuring emission at 510 nm (Fluoroskan Ascent FL, Labsystems, Finland). Briefly, effector cells were plated and treated with MMC. Target cells were co-plated and bystander cytotoxicity was measured as described previously [18]. Wherever medium transfer experiments were performed, the medium was supplemented with 0.2% FBS to avoid cell death due to growth factor depletion.

### MTT assay to evaluate bystander cytotoxicity in co-cultures containing effector and target Hep3B cells

Effector Hep3B cells were plated (3.5 × 10^3 ^and 7.5 × 10^3 ^cells per well) in a 96 well plate and allowed to adhere for 24 h at 37°C. Cells were treated with MMC for 24 h. Subsequently, effector cells were washed twice with medium and target Hep3B cells (7.5 × 10^3 ^cells per well) were co-plated. After 72 h medium was decanted and MTT assay was performed.

### Trypan blue dye exclusion assay to detect involvement of TRAIL in mediating MMC induced bystander effect in co-cultures containing effector and target Hep3B cells

Hep3B cells (2 × 10^5 ^cells per well) were plated in a six well plate and allowed to adhere for 24 h at 37°C. Cells were treated with MMC for 24 h. Thereafter, effector cells were washed twice with medium and target Hep3B cells (2 × 10^5 ^cells per well) were co-plated with or without neutralizing anti-TRAIL antibody. After 72 h of growth in co-culture, trypan blue dye exclusion assay was performed as described previously [20].

### Conditioned medium collection and sandwich ELISA for detection of secreted FasL and TRAIL from effector cells

Secreted FasL and TRAIL were detected in conditioned medium (CM) by sandwich ELISA. To obtain CM, 7 × 10^5 ^effector cells were plated in 60 mm tissue culture dishes and kept overnight for adherence. Cells were treated with MMC (150 nM) for 24 h. Wherever EDTA was used, cells were pre-treated with 2 μM EDTA for 1 h and MMC treatment was given in the presence of EDTA for 24 h. Subsequently, medium was decanted, cells were washed twice with medium and phenol-red free DMEM without FBS was added to the cells. Cells were cultured for additional 24 h, 48 h, 72 h and 96 h. After each time period CM was collected, centrifuged at 50,000 × g for 30 min and supernatants were concentrated to half of the original volume by SpeedVac (Thermo Savant, USA). For ELISA, each well of micro-titre plate was coated with 50 μl of anti-FasL Mab and/or anti-TRAIL Mab (250 ng/50 μl) for 3 h at 37°C, followed by blocking of free sites with 100 μl of blocking reagent (5% BSA in PBS) for 2 h at 37°C. Test samples (50 μl) were added and incubated for 3 h at 37°C followed by incubation with 50 μl of second anti-FasL and/or anti-TRAIL polyclonal antibody, for 3 h at 37°C. After that, secondary HRP conjugated antibody was added in each well. Finally, ABTS in 0.1 M Na-citrate buffer (pH 4.0) was added to each well and incubated for 10 min at room temperature before taking absorbance at 405 nm. Five washes with PBS containing 0.05% Tween-20 were performed between each step and plates were pat dried.

### Western blot analysis and immunostaining

Whole cell lysates were made and western blotting was performed as described previously [18]. For immunostaining, cells were fixed, permeabilized and immunofluorescence was performed as described earlier [19].

### Bystander cell killing in soft-agarose colony formation assay

Base agarose layer (1%) was made in a tissue culture plate and a top agarose layer (0.8%) containing HepG2 cell was added over the base layer. Consequently, plates were kept in incubator and cells were allowed to grow inside the semisolid agarose containing culture medium. After 4 weeks when sizable colonies appeared, a center well was made in plates with the help of micropipette tip in which untreated cells were added in control plate and treated cells were added in the experimental plate. These wells were covered with 0.8% agarose. The plates were incubated for additional 4-5 weeks. After that colonies in both plates were counted manually under microscope and pictures were taken. The mean diameter of colonies was calculated using ImagePro Plus 5.0 software (MediaCybernetics, Silver Spring, MD, USA). Data thus obtained on number of colonies and size of colonies was depicted by graph.

### Detection of apoptosis in effector and target cells by TUNEL assay

TdT mediated dUTP Nick End Labeling (TUNEL) assay was performed by using APO-DIRECT (BD Biosciences, CA, USA) and/or by In-situ cell death detection kit (Roche Applied Science, PA, USA) following the manufacturer's protocol. Briefly, HepG2 and Hep3B cells (7 × 10^3^) were seeded in multi-well slides (MP Biomedicals, OH, USA). For effector cells, MMC treatment was given for 24 h with the indicated concentration. Thereafter, drug containing medium was decanted and fresh medium without MMC was added to each well. Cells were cultured in drug free medium for additional 72 h. In case of target cells medium transfer experiment was performed. Effector cells (5 × 10^4^) were plated in a 35 mm tissue culture dish and treated with indicated concentration of MMC for 24 h. Following treatment the effector cells were washed twice with medium and fresh medium without drug was added to the cells and incubated for additional 48 h. The medium thus obtained was utilized to culture target cells for 48 h before performing TUNEL assay. The medium was supplemented with 0.2% FBS to avoid growth factor depletion. For TUNEL assay cells were washed with PBS and fixed with 3.7% paraformaldehyde, permeabilized with 0.1% Triton X-100 and reaction mixture containing TdT enzyme, FITC labeled dUTP was added to cells. The reaction was performed at 37°C for 1 h. Cells were then washed with rinsing buffer, PI solution was added to each well and kept at 37°C for 30 min. After washing cells thoroughly they were overlaid with mounting medium containing antifade (Santa Cruz Biotechnology, CA, USA). The slides were subjected to confocal microscopy (Zeiss LSM510, Heidelberg, Germany). Images were processed by Adobe Photoshop software. Wherever In-situ cell death detection kit was used to detect apoptosis, PI (50 μg/ml) was added to the cells and incubated at 37°C for 30 min before they were overlaid with mounting medium.

### Statistical analysis

The data were calculated with sigma plot 10 and values are presented as arithmetic mean ± standard deviation (mean ± s.d). Unpaired student's t-test was used to determine the statistical significance of differences between samples and p-value < 0.05 were considered significant. * is for p-value < 0.05, ** is for p-value < 0.005 and *** is for p-value < 0.0005.

## Results

### Growth pattern and chemosensitivity of EGFP expressing cells was similar to their corresponding parental cells

**Figure 1:**
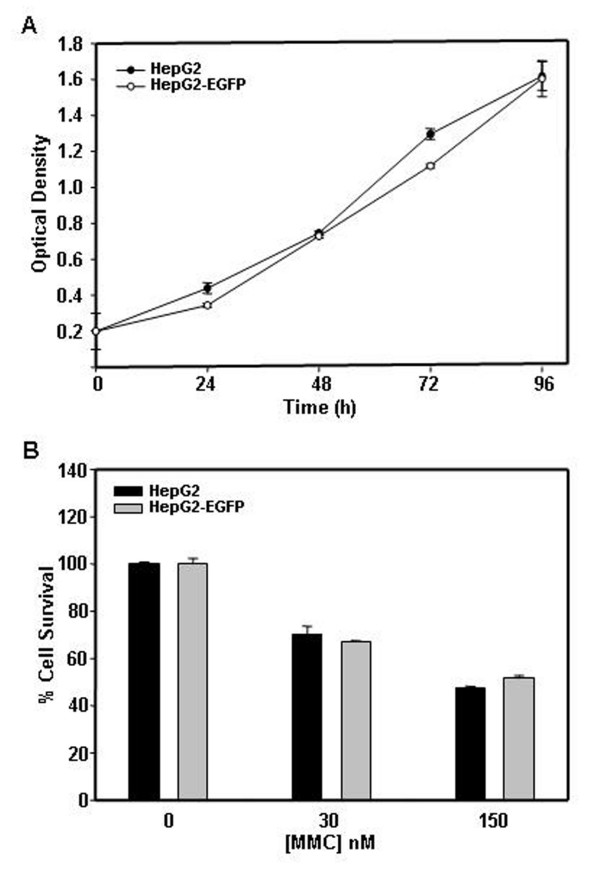
**Cell growth and cytotoxicity assay in HepG2 and HepG2-EGFP cell lines**. (A) Growth kinetics of HepG2 and HepG2-EGFP cells shows no significant alteration in growth properties. HepG2 and HepG2-EGFP cells (2 × 10^3^) were seeded in a 96 well plate and subsequently cultured for 24 h, 48 h, 72 h and 96 h, and MTT assay was performed. Absorbance was measured at 570 nm using 630 nm as reference filter. Optical density of 0.2 corresponds to 2 × 10^3 ^cells. (B) MMC is equally cytotoxic to HepG2 and HepG2-EGFP cells. HepG2 and HepG2-EGFP cells (1 × 10^4^) were seeded in a 96 well plate and kept overnight for adherence. MMC treatment with indicated concentrations was given for 48 h. Cells were grown in a drug free medium for 24 h before performing the MTT assay. Data presented are representative of three independent experiments performed in triplicates and expressed as mean ± s.d.

**Figure 2:**
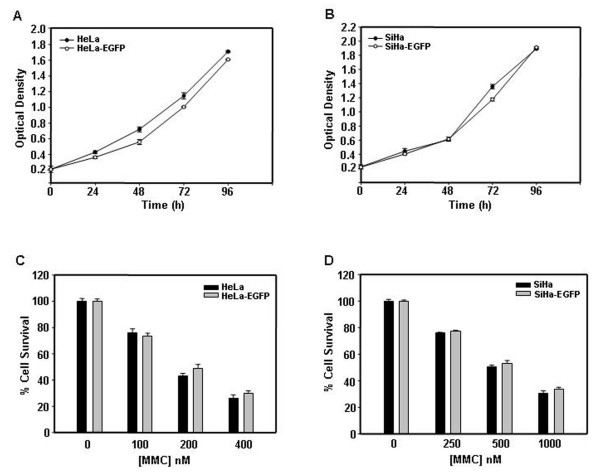
**Cell growth and cytotoxicity assay for HeLa/HeLa-EGFP cells and for SiHa/SiHa-EGFP cells**. Cells (2 × 10^3^) were seeded in a 96 well plate and subsequently cultured for 24 h, 48 h, 72 h and 96 h and MTT assay was performed. Absorbance was measured at 570 nm using 630 nm as reference filter. Optical density of 0.2 corresponds to 2 × 10^3 ^cells. (A) Growth kinetics of HeLa and HeLa-EGFP cells. (B) Growth kinetics of SiHa and SiHa-EGFP cells. (C) MMC cytotoxicity on HeLa and HeLa-EGFP cells. (D) MMC cytotoxicity on SiHa and SiHa-EGFP cells. Cytotoxicity assay for HeLa, HeLa-EGFP, SiHa and SiHa-EGFP was performed as done for HepG2 and HepG2-EGFP. Data presented are representative of three independent experiments performed in triplicates and expressed as mean ± s.d.

To investigate the growth properties and chemosensitivity of parental and EGFP expressing cells, we performed an MTT assay. Growth pattern of HepG2 and HepG2-EGFP cells were similar (Fig. 1A). Both HepG2 and HepG2 EGFP cells were equally sensitive to MMC treatment (Fig. 1B). EGFP expressing HeLa and SiHa cells were also found to be similar to parental cells in their growth pattern as well as chemosensitivity towards MMC (Fig. 2 panel A to D).

### MMC induced bystander cytotoxicity in hepatoma cells is proportional to drug dosage

**Figure 3:**
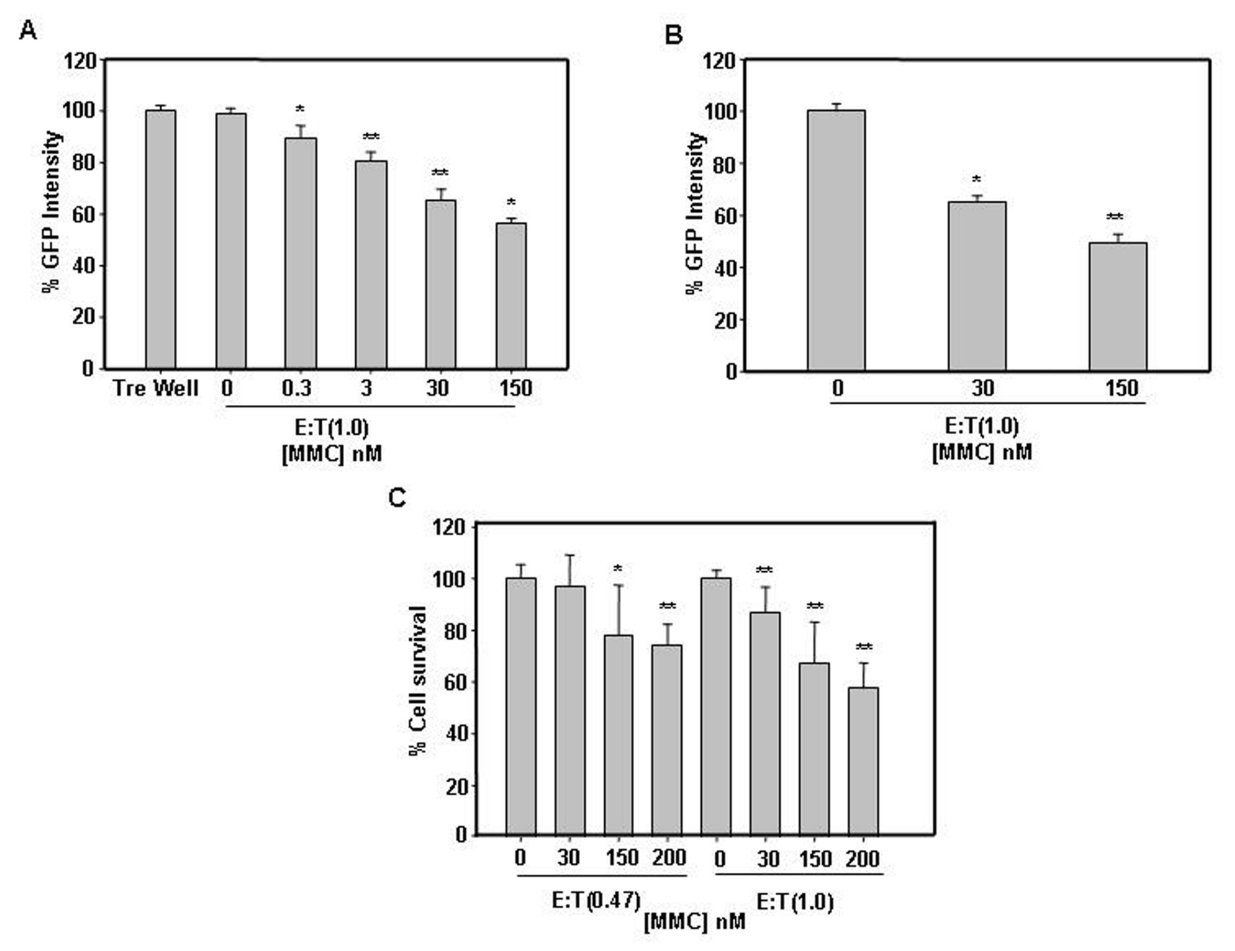
**Co-culture experiments to evaluate MMC induced bystander killing**. (A) Bystander effect mediated by effector HepG2 cells treated with MMC (0.3 nM, 3 nM, 30 nM and 150 nM) for 24 h, towards target HepG2-EGFP cells. Effector cells were plated in a 12-well culture plate at a density of 5 × 10^4 ^cells per well and kept for adherence at 37°C for 24 h. MMC treatment was given for 24 h, after that medium was decanted and cells were washed twice with medium. 5 × 10^4 ^target cells were co-plated and the co-cultures were allowed to grow at 37°C for 72 h. Subsequently cells were trypsinized and total live fluorescence was quantified. In order to avoid any possibility of cell death due to residual drug in the well, we used an empty well containing no effector cells but only medium containing 150 nM MMC and subsequently this well was processed similarly before plating the target HepG2-EGFP cells. (B) Bystander effect mediated by effector Hep3B cells treated with MMC (30 nM and 150 nM) towards target HepG2-EGFP cells. (C) Bystander effect mediated by effector Hep3B cells towards target Hep3B cells. MTT assay was performed to evaluate bystander cytotoxicity in Hep3B cells. Cells were plated and treated with MMC (30 nM, 150 nM and 200 nM) for 24 h and target Hep3B cells were co-plated as described in materials and methods. After 72 h medium was decanted and 50 μl MTT (1 mg/ml) was added in each well and incubated for 4 h at 37°C. Subsequently, formazan crystals were solubilized in 50 μl of iso-propanol by incubating with shaking for 10 min, at room temperature. Absorbance was measured at 570 nm using 630 nm as reference filter. Data presented are representative of three independent experiments performed in triplicates and expressed as mean ± s.d. * and ** significantly differs from there respective controls.

*In vitro *co-culture experiments were performed to investigate the induction of bystander effect in hepatoma cells. Briefly, effector cells were treated with MMC and thereafter target cells were co-plated. MMC treated HepG2 and Hep3B cells induced bystander killing of unexposed cells as determined by measuring total live fluorescence of HepG2-EGFP in the target cells (Fig. 3A and Fig. 3B). MMC treated Hep3B cells also induced bystander killing in unexposed Hep3B cells as determined by measuring cell survival by MTT (Fig. 3C). The bystander killing of target cells was directly proportional to the drug dose with which the effector cells were treated. The cell survival decreased with increasing concentration of MMC.

### Bystander cytotoxicity is not observed in co-cultured cervical cancer cells treated with MMC

**Figure 4:**
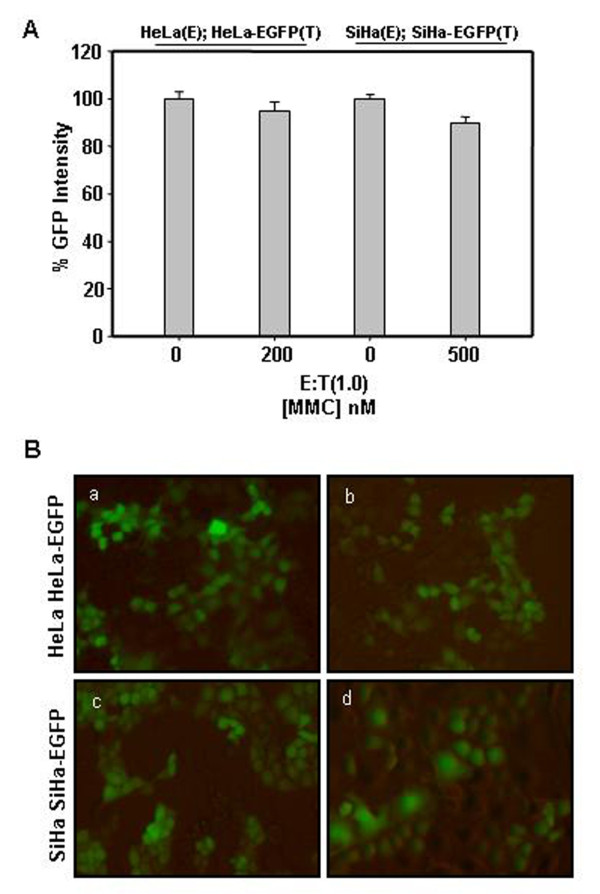
**MMC fails to induce bystander killing in cervical cancer cell**. (A) Effector HeLa and SiHa cells were plated and treated with MMC (200 nM and 500 nM respectively) for 24 h. EGFP expressing target HeLa and SiHa cells were co-plated and the co-culture were allowed to grow for 72 h after that cells were trypsinized and total live fluorescence was quantified. Data presented are representative of three independent experiments and are expressed as mean+s.d. of triplicate samples. (B) Fluorescent photomicrographs of co-cultured cells to evaluate MMC induced bystander killing: (a) untreated HeLa cells co-plated with HeLa-EGFP, (b) MMC treated HeLa cells (200 nM) co-plated with HeLa-EGFP, (c) untreated SiHa cells co-plated SiHa-EGFP, (e) MMC treated SiHa cells (500 nM) co-plated with SiHa-EGFP.

In an attempt to determine does MMC also cause bystander effect in other solid tumor cells, HeLa and SiHa effector cells were treated with MMC and corresponding target cells stably expressing EGFP were co-plated. Though, MMC per se was toxic to cervical cancer cells, no cell killing by bystander effect was observed, as determined by measuring total live fluorescence (Fig. 4A) and also as depicted in fluorescent photomicrographs (Fig. 4B).

### MMC induced bystander effect is transferable through medium and extent of killing depends upon the number of effector cells

**Figure 5:**
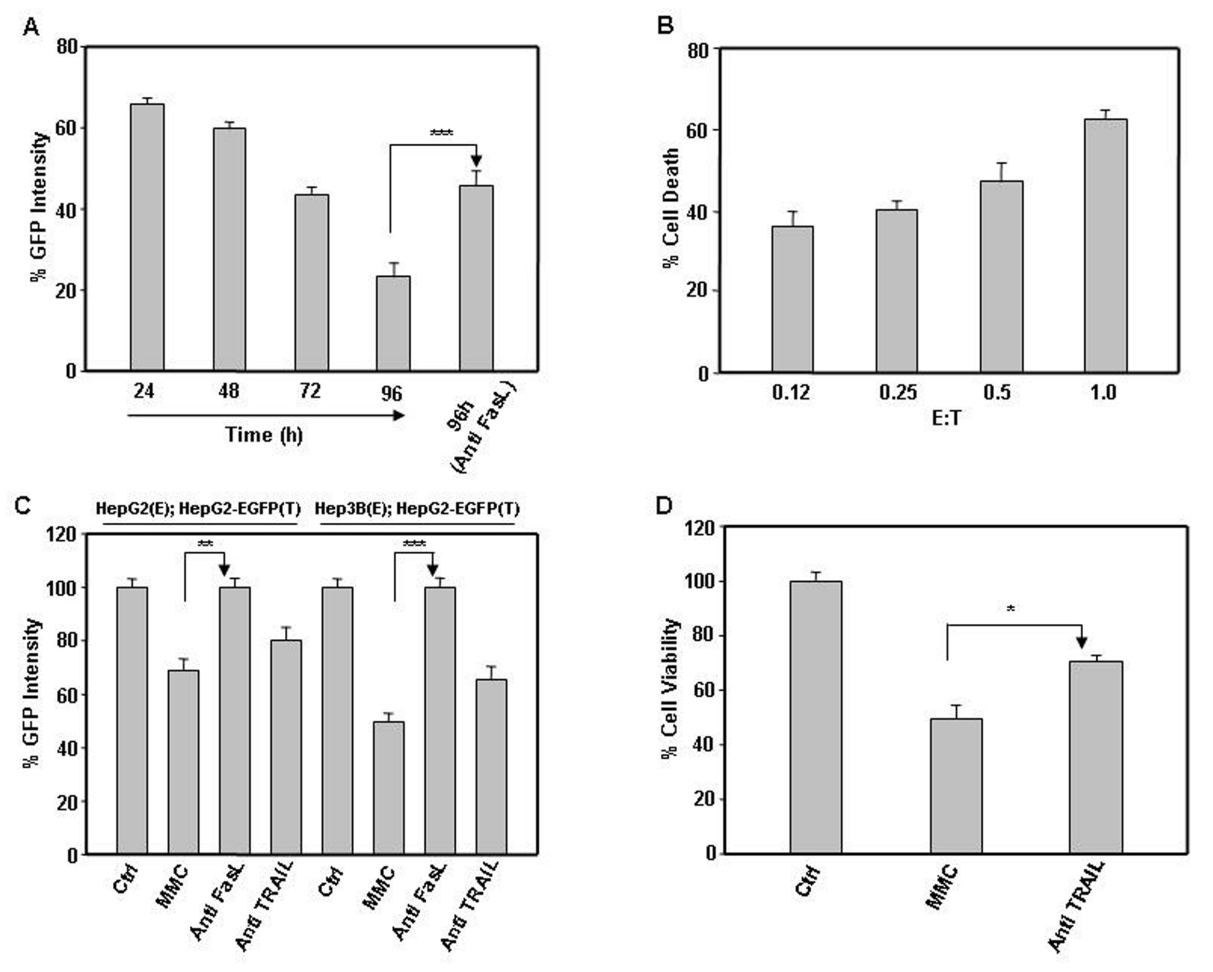
**Medium transfer experiments to detect the presence of soluble cytotoxic effector molecules**. (A) Time-course release of cytotoxic factor from MMC treated effector HepG2 cells. The effector cells were treated with MMC for 24 h and fresh medium was added as described in materials and methods. At the time indicated, corresponding supernatants were collected and added to the untreated target HepG2-EGFP cells with or without neutralizing anti-FasL antibody. Target cells were incubated with the supernatants for 48 h before quantitating fluorescence. The supernatants were supplemented with 0.2% FBS to avoid cell death due to growth factor depletion. The culture supernatant from untreated HepG2 cells was used as control and fluorescent intensity was calculated with respect to identical time point controls. (B) The target cell death increases with the increase in number of effector cells. Effector cells were treated with MMC for 24 h and fresh medium was added as described in materials and methods. The supernatants were collected after 48 h and used to culture target HepG2-EGFP cells for 48 h before quantitating fluorescence. (C) Involvement of death ligands in bystander killing of hepatoma cells. Bystander killing of HepG2-EGFP cells is mediated by FasL. Effector HepG2 and Hep3B cells were treated with MMC (150 nM) and target HepG2-EGFP cells were co-plated with or without neutralizing antibody against FasL (1 μg/ml) and TRAIL (2 μg/ml). (D) Bystander killing of target Hep3B cells by effector Hep3B cells treated with MMC (150 nM) is mediated by TRAIL. Trypan blue assay to detect the percentage cell viability in co-cultures of effector and target Hep3B cells in the presence of neutralizing anti-TRAIL antibody. After 72 h of growth in co-culture cells were harvested by trypsinization and stained with 0.005% trypan blue for 5 min at RT. Unstained as well as stained cells were counted in haemocytometer and percentage cell viability was calculated. Data presented are representative of three independent experiments performed in triplicates and expressed as mean ± s.d. *, ** and *** differs significantly at p < 0.05.

To investigate whether soluble toxic factors are involved in killing of target cells by bystander effect, we performed medium transfer experiments. Medium collected from cells treated with MMC for different time duration was used for culturing untreated HepG2-EGFP cells. After 48 h, total live fluorescence was quantified as mentioned in materials and methods. The fluorescence intensity significantly decreased in cells cultured in medium obtained from MMC treated cells. The decrease in fluorescence intensity was proportional to the time after which medium was collected from the MMC treated cells. Moreover, there was an increase in fluorescence intensity when neutralizing anti-FasL antibody was added in the medium transferred onto the target cells at 96 h time point (Fig. 5A). Furthermore, bystander killing also increased with an increase in the ratio of effector to target cells and maximum cell death was observed when this ratio was one (Fig. 5B).

### Both membrane bound and secreted form of death ligands are involved in MMC induced bystander effect

**Figure 6:**
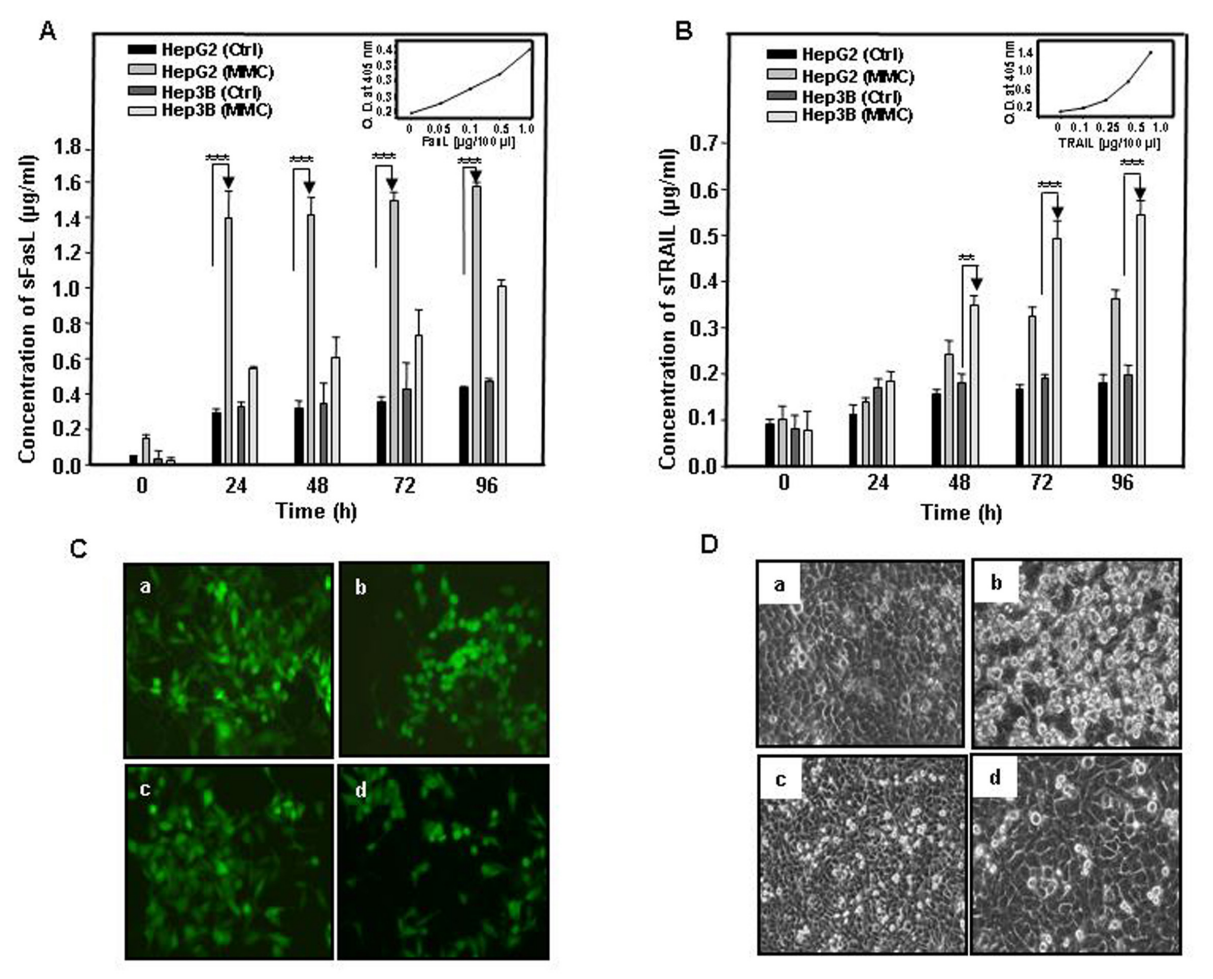
**Sandwich ELISA to detect secreted form of death ligands in conditioned medium obtained from MMC treated hepatoma cells**. (A) Sandwich ELISA for quantification of secreted FasL from untreated and treated HepG2 and Hep3B cells at different time points. (B) Sandwich ELISA for quantification of secreted TRAIL from untreated and treated HepG2 and Hep3B cells at different time points. Data presented are representative of three independent experiments performed in triplicates expressed as mean ± s.d. *** differs significantly from the respective control. (C) Fluorescent photomicrographs of co-cultured cells to evaluate MMC induced bystander killing of HepG2-EGFP cells: (a) untreated effector HepG2 cells, (b) effector cells treated with MMC (150 nM), (c) neutralizing anti-FasL antibody (1 μg/ml), (d) neutralizing anti-TRAIL antibody (2 μg/ml). (D) Photomicrographs of co-culture of effector and target Hep3B cells: (a) untreated effector Hep3B cells with target Hep3B cells, (b) MMC (150 nM) treated effector Hep3B cells with target Hep3B cells, (c) effector Hep3B cells treated with MMC and target Hep3B cells co-plated in the presence of neutralizing anti-TRAIL antibody (2 μg/ml), (d) effector Hep3B cells pretreated with EDTA (2 μM) followed by MMC treatment and then target Hep3B cells were co-plated.

To explore the involvement of death ligands as a facilitator of cell killing by bystander effect, we utilized neutralizing antibodies against FasL and TRAIL and also detected the presence of secreted forms of FasL and TRAIL in CM by sandwich ELISA. It is already reported that chemotherapeutic drugs up- regulate the expression of FasL and TRAIL [21] and in several studies the involvement of these death ligands in bystander effect has been reported [18,22-24]. When the EGFP expressing target HepG2 cells were co-plated with MMC treated effector cells (HepG2 and Hep3B) in the presence of neutralizing anti-FasL antibody, the EGFP intensity of target cells was similar to that of cells co-plated with untreated cells. Under similar experimental conditions, when neutralizing anti-TRAIL antibody was utilized, no significant change in the EGFP intensity of target cells was observed. On the other hand, in mixed co-cultures of effector Hep3B and target HepG2-EGFP in the presence of neutralizing anti-TRAIL antibody, cell survival was not altered, whereas in the presence of neutralizing anti-FasL antibody the cell survival increased significantly (Fig. 5C). In co-cultures containing effector Hep3B and target Hep3B cells in the presence of neutralizing anti-TRAIL antibody, cell survival increased in comparison to cells which were not incubated with the antibody, as determined by measuring percentage cell viability by trypan blue exclusion assay (Fig. 5D). The presence of the secreted form of these ligands in CM of MMC treated cells was detected by sandwich ELISA. There was a significant increase in FasL in CM obtained from treated HepG2 cells as compared to CM from control cells taken at identical time points. Unlike in HepG2 cells, there was significant increase in FasL and TRAIL in CM obtained from treated Hep3B cells in comparison to CM from control cells taken at identical time points (Fig. 6A and 6B). The levels of secreted ligands increased up to 96 h following MMC treatment. The amount of FasL released from treated HepG2 cells was in the range of 1.4 μg/ml to 1.6 μg/ml from 7 × 10^5 ^cells, which was significantly more than the amount of TRAIL released (0.15 μg/ml to 0.36 μg/ml). The amount of TRAIL released by 7 × 10^5 ^Hep3B cells ranged between 0.18 μg/ml to 0.54 μg/ml. Finally, the involvement of FasL and TRAIL was confirmed by utilizing respective neutralizing antibodies, as shown in fluorescent photomicrographs of HepG2 cells (Fig. 6C) and Hep3B cells (Fig. 6D). Collectively, these results indicate the involvement of FasL in transferring cytotoxicity in HepG2 cells. In Hep3B cells, though MMC treatment facilitated release of both FasL and TRAIL, due to reduced expression of Fas in Hep3B [21], the secreted FasL did not promote toxicity and instead secreted TRAIL was involved in propagating bystander cell killing following MMC treatment.

### Expression of death receptors increases following MMC treatment

**Figure 7:**
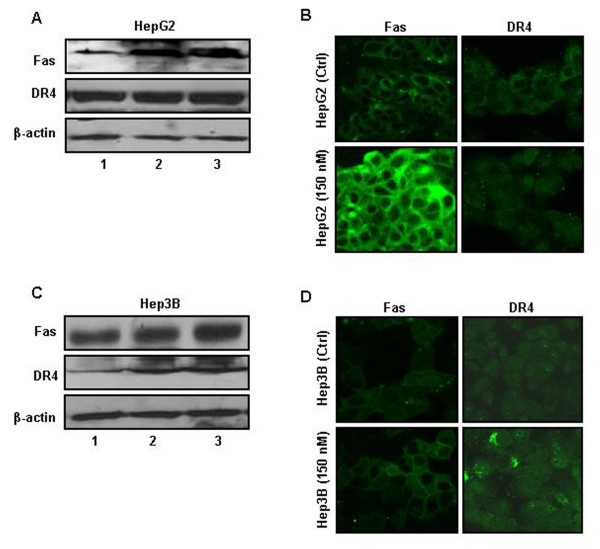
**Expression of death receptors increases after MMC treatment in hepatoma cells**. HepG2 and Hep3B cells were treated with MMC for 24 h. Cells were washed twice with medium, fresh medium without MMC was added and cells were incubated again for 24 h at 37°C. After post-treatment growth in MMC free medium whole cell lysate was prepared and western blot was performed. (A) Western blot analysis: lane (1) control HepG2 cells, lane (2) HepG2 treated for 24 h with MMC and lane (3) HepG2 treated for 24 h with MMC followed by post treatment growth in drug free medium for 24 h. β-actin was detected as a loading control. Where ever required, blots were stripped by incubating the membranes at 50°C for 30 min in stripping buffer (62.5 mM Tris-Cl pH 6.7, 100 mM mercaptoethanol, 2% SDS) with intermittent shaking. Membranes were washed thoroughly with TBS and reprobed with required antibodies. Otherwise gels run in duplicates were probed for the desired proteins by western blotting. (B) Immunofluorescence staining of HepG2 cells. Cells were treated with MMC for 24 h, washed twice with medium and fresh medium without MMC was added and cultured for 24 h. Cells were fixed with 4% paraformaldehyde, permeabilized with 1% Triton X- 100 and blocked with 5% FBS. Cells were then incubated with anti-FasL, anti-Fas, anti-TRAIL and anti-DR4 primary antibodies for 2 h and subsequently stained with FITC conjugated secondary antibody for 1 h.

Drug treatment, in addition to facilitating an increase of death ligands, has also been shown to enhance the expression of their cognate receptors on the cell membrane [25]. Therefore, to ascertain that MMC treatment also alters the expression of death receptors, we examined the expression of Fas and TRAIL receptors after MMC treatment. It was observed that the expression of Fas receptor increased in HepG2 cells, but no change in TRAIL receptor DR4 was detected (Figure 7A). The expression of TRAIL receptor DR4 increased significantly in Hep3B cells, but no changes in Fas receptor were observed (Figure 7C). These results are also supported by confocal laser scanning images of the cells (Fig. 7B and 7D).

### Treatment with EDTA decreases bystander cytotoxicity

**Figure 8:**
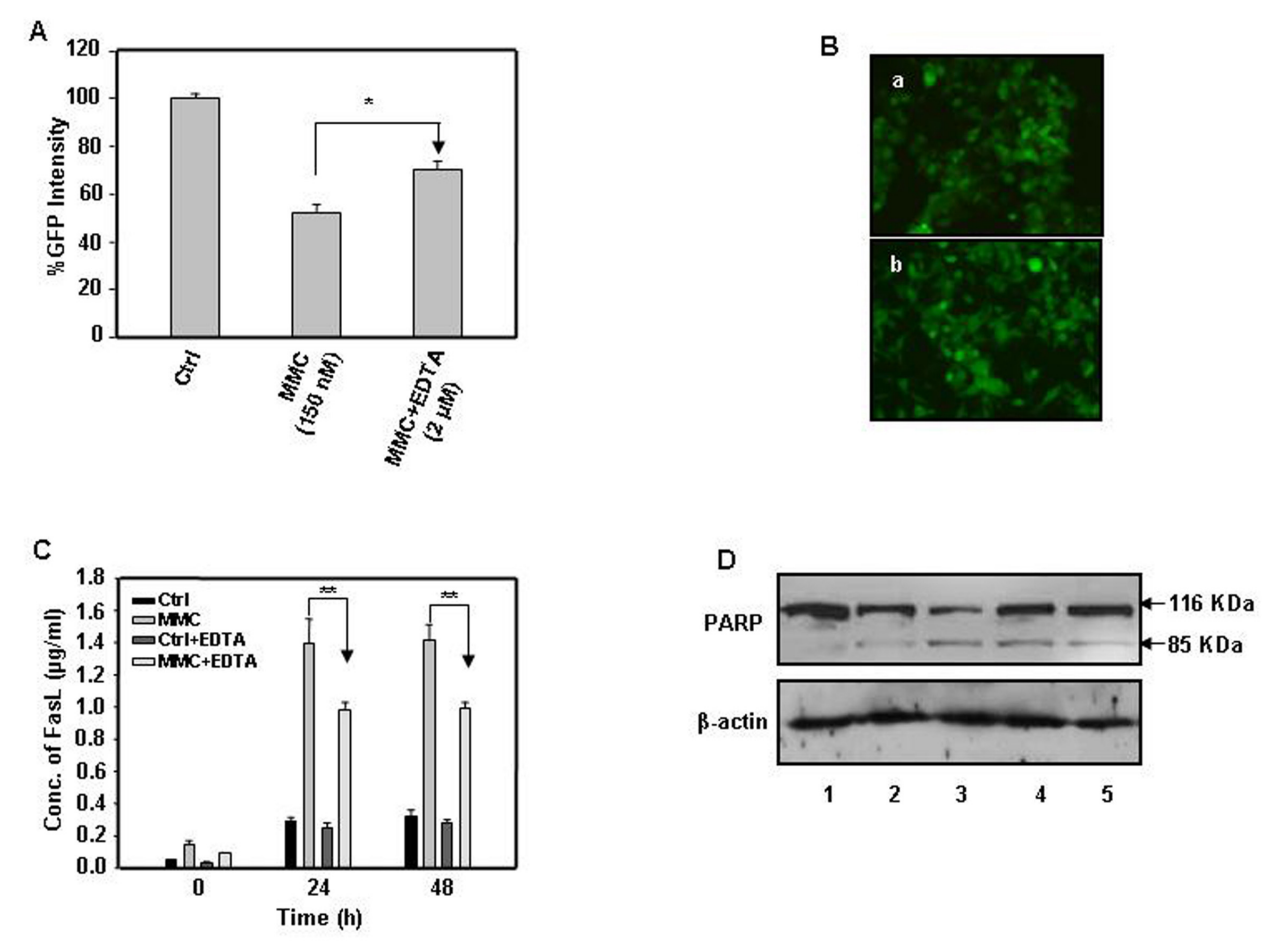
**Secreted form of FasL involved in mediating bystander cytotoxicity and apoptosis in HepG2 cells**. (A) Effector HepG2 cells were pretreated with EDTA (2 μM) for 1 h and MMC was added in the same medium containing EDTA. Subsequently, this medium was used to culture target HepG2-EGFP cells and fluorescent intensity was measured after 48 h. (B) Fluorescent photomicrograph: (a) untreated HepG2 effector cells co-plated with target HepG2-EGFP cells and (b) effector HepG2 cells pretreated with 2 μM EDTA followed by MMC treatment for 24 h. (C) Sandwich ELISA to detect FasL in CM from MMC treated and cells pretreated with EDTA followed by MMC treatment for 24 h. Data presented are representative of three independent experiments performed in triplicates and expressed as mean ± s.d. ** differs significantly at p < 0.005. (D) Effector HepG2 cells treated with MMC for 24 h as well as from effector HepG2 cells pretreated with EDTA for 1 h followed by MMC treatment for 24 h. Cells were then washed twice with medium, target HepG2-EGFP cells were co-plated and the co-culture were grown for 72 h, and whole cell lysates were made from the co-culture to perform western blot analysis. The levels of PARP p116 proform and its cleavage product p85 were detected. β-actin was detected as a loading control. Lane (1) untreated effector HepG2 cells. (2) MMC treated effector cell and harvested after 24 h. (3) MMC treated effector cells and harvested after 48 h. (4) EDTA (2 μM) pre-treatment followed by MMC treatment of effector cells and harvested after 24 h. (5) EDTA (2 μM) pre-treatment followed by MMC treatment of effector cells and harvested after 48 h.

The involvement of secreted death ligands in MMC induced bystander effect was further confirmed by utilizing EDTA, a broad range protease inhibitor. EDTA inhibits the cleavage of FasL from its membrane [26]. In the presence of EDTA, bystander killing of target cells decreased as evident by an increase *in *total live fluorescence (Fig. 8A) and also as shown in fluorescent photomicrographs of HepG2 cells (Fig. 8B) and Hep3B cells (Fig. 6D panel a, b and d). Furthermore, to verify whether EDTA treatment caused a decrease in release of secreted FasL from MMC treated HepG2 cells, we performed sandwich ELISA in CM obtained from treated effector HepG2 cells and the cells pre-treated with EDTA followed by MMC treatment. There was a significant decrease in the quantity of FasL released from the cells pre-treated with EDTA followed by MMC treatment as compared to only MMC treated cells (Fig. 8C). This result was also supported by western blot analysis of CM from MMC treated HepG2 cells as well as from cells pre-treated with EDTA followed by MMC treatment (Additional file 1). To determine whether the cytotoxicity mediated by MMC was due to induction of apoptosis, we detected PARP cleavage by western blotting in co-cultures containing MMC treated effector HepG2 cells co-plated with target HepG2-EGFP cells, in the presence and absence of EDTA. PARP cleavage decreased in MMC treated cells pre-exposed to EDTA (Fig. 8D).

### Both effector and target cells undergo apoptosis following MMC treatment

**Figure 9:**
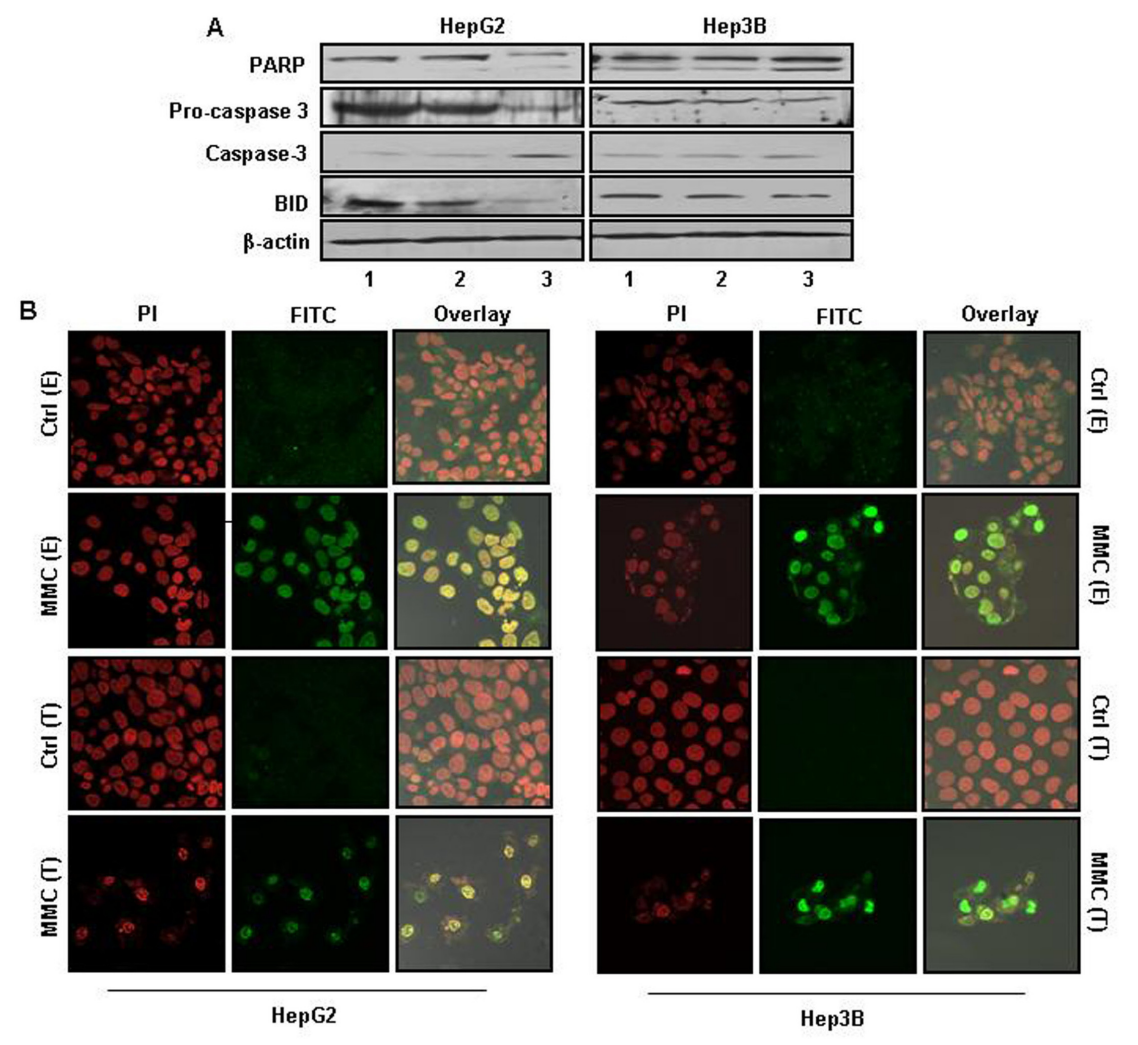
**MMC induced apoptotic factors**. (A) HepG2 and Hep3B cells treated with MMC were harvested and cell lysates were prepared. The expression of apoptosis related proteins were dectected by western blot analysis. The protein levels of PARP p116 proform and its p85 cleavage product, pro caspase-3 p32 and its cleavage product p11, and BID p21 proform were detected. Lane (1) untreated cells, (2) cells treated with MMC for 24 h and (3) cells treated with MMC for 24 h followed by post treatment growth in MMC free medium for 24 h. β-actin was detected as a as loading control. Where ever possible blots were stripped and reprobed with required antibodies. Otherwise gels run in duplicates were probed for the desired proteins by western blotting. (B) TUNEL assay for effector and target cells. For effector cells, HepG2 and Hep3B cells were treated with MMC (150 nM) for 24 h. Then the cells were washed with medium and cultured for additional 72 h before performing TUNEL assay. Similarly, for target cells, medium form untreated as well as from treated HepG2 or Hep3B cells were used to culture target HepG2 and Hep3B cells respectively for 48 h before performing TUNEL assay. The medium was supplemented with 0.2% FBS to avoid growth factor depletion.

We examined the expression of apoptosis related molecules in both HepG2 and Hep3B cells after MMC treatment. Decreased expression of PARP p116, pro caspase-3, BID and increased levels of caspase-3 was observed in MMC treated cells (Fig. 9A). This observation was further supported by TUNEL assays in which HepG2 and Hep3B cells were treated with MMC for 24 h and then cultured in drug free medium for an additional 72 h. Also, the effect on the target cells was investigated by transferring CM from the treated effector cells and incubating them for 48 h before performing a TUNEL assay. Results from all of these experiments clearly demonstrated that MMC induced apoptosis directly in the effector cells and by bystander effect in the target cells (Fig. 9B).

### Death factors released by MMC treated cells significantly reduce the size of colonies in soft-agarose assay

**Figure 10:**
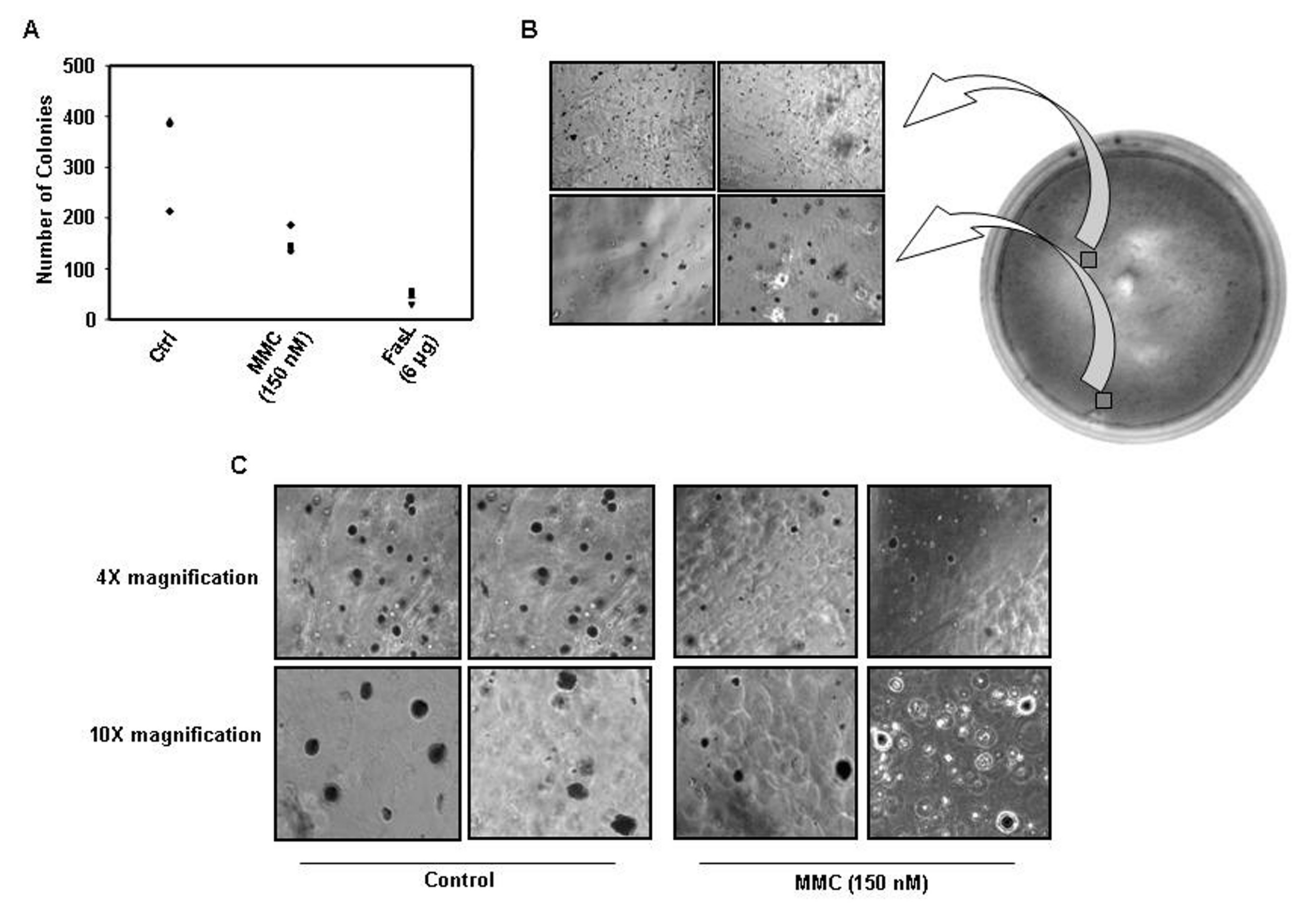
**Soft-agarose colony formation assays to evaluate the role of secreted FasL in mediating cytotoxic effect**. Base agarose layer was made in DMEM without phenol red and top agarose layer containing 4 × 10^3 ^HepG2 cells was poured over the base layer. After 4 weeks of growth in semisolid medium untreated and treated effector cells (5 × 10^5^) were added in the center well of the plates. These plates were incubated at 37°C for 4 weeks. (A) Number of colonies decreased in the presence of effector cells treated with MMC as compared to control effector cells. The number of colonies decreased drastically in the presence of recombinant FasL (6 μg) which served as a positive control. (B) Photomicrographs of soft-agarose plate containing MMC treated effector HepG2 cells. The colony size diminished and very small colonies (<20 μm in mean diameter) were observed in the area surrounding center well containing treated effector cells. (C) Photomicrographs of soft-agarose colonies at 4× magnification and 10× magnification corresponding to different fields from the plate containing control effector cells and the plate containing MMC treated effector cells, in the center well.

**Figure 11:**
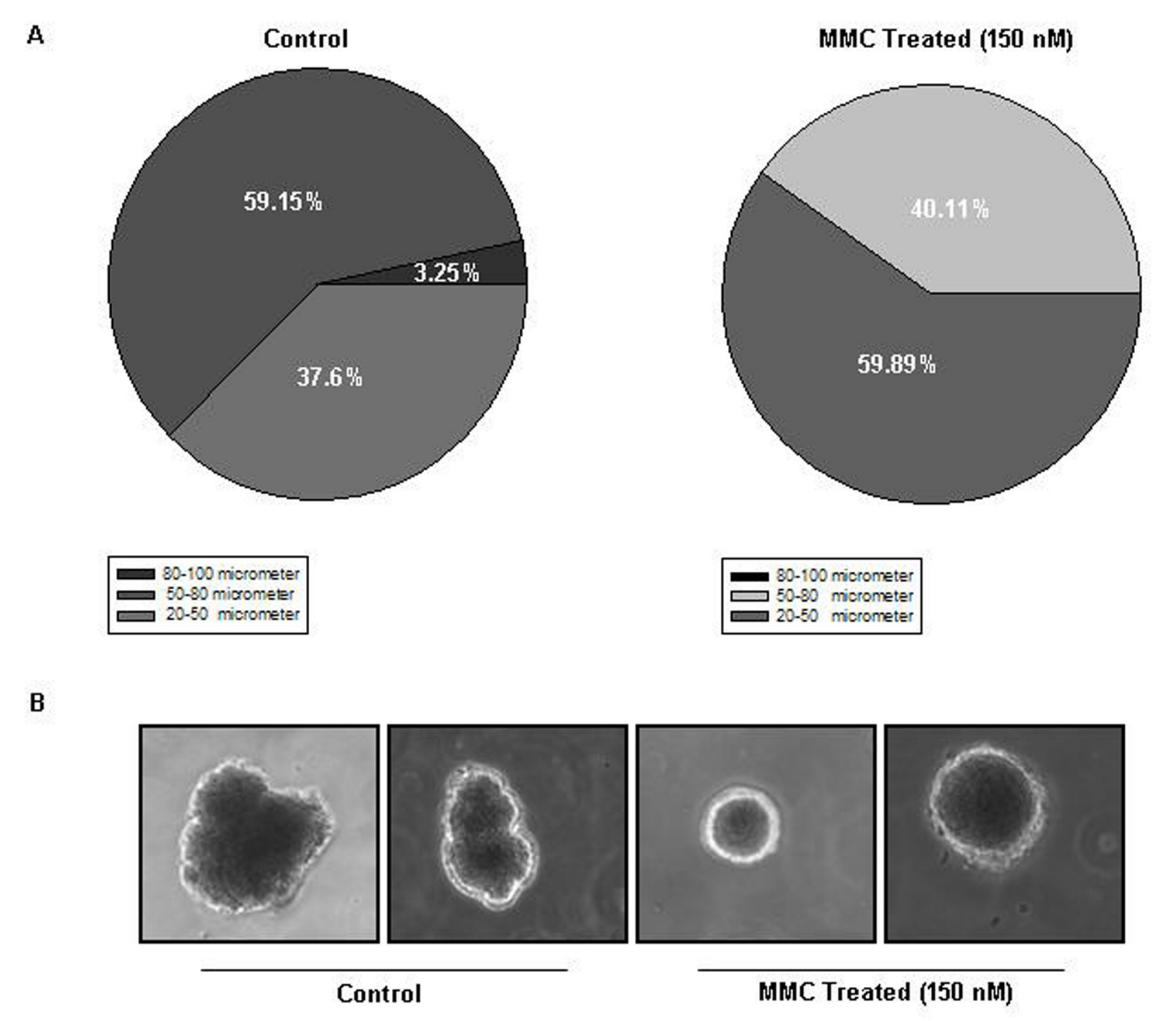
**Release of secreted cytotoxic factors diminishes considerably the size of soft-agarose colonies**. (A) Pie chart showing percentage distribution of colonies of different mean diameters (colonies >20 μm in diameter were considered) in the soft-agaose plate containing untreated effector HepG2 cells and in the plate containing MMC treated effector HepG2 cells. (B) Photomicrographs of colonies at higher magnification showing diminished growth when MMC treated effector HepG2 cells were added in the center well of soft-agarose plate containing colonies of target cells.

Because the bystander cell killing was transferable via medium, we designed a novel experimental strategy to ascertain that this effect was also transferable through solid medium. We took advantage of soft-agarose colony formation assay in which cells not only grow as a mass to mimic tumor, but also represent anchorage independent growth of tumors. Death inducing soluble factors secreted by the effector cells are likely to diffuse slowly over a period of time. This diffusion of death signal, if effective, would have an impact on either the number or size of colonies during the incubation period of 4 to 5 weeks after seeding of effector cells. Interestingly, colony size of the target cells in which untreated effector cells were seeded and allowed to grow varied significantly in mean diameter, most of them ranging between 50-80 μm. On the other hand, the colony size of the target cells in which treated effector cells were seeded was reduced dramatically, with most colonies ranging between 20-50 μm. Also, the number of colonies was significantly less as compared to those in a plate containing untreated effector cells (Fig. 10 and Fig. 11). Almost no colonies were detected in plates in which pure recombinant FasL was added in the centre well. When the treated HepG2 effector cells together with a neutralizing anti-FasL antibody were added in the center well, more colonies were detected (Additional file 2C and 2D). This result was further supported by yet another colony formation assay in which medium from untreated and/or treated effector HepG2 cells was added in the plates containing target HepG2 cells in either the absence or presence of the neutralizing anti-FasL antibody. The number of colonies present was very low in the plates in which medium from treated effector cells was added in comparison to the colonies present in the plates in which medium from untreated cells or in which medium supplemented with neutralizing anti-FasL antibody was added (Additional file 3). These results clearly demonstrate that MMC induced release of FasL is able to propagate its effect at a distinct location.

## Discussion

In the present study we addressed the efficacy of MMC in hepatoma cells by exploiting its ability to promote bystander killing in addition to its direct toxicity. Our results from the co-culture experiments indicated that the bystander signal initiated after 24 h of treatment, even at a low dose of 0.3 nM MMC. Significant increase in bystander killing was observed up to 72 h after drug treatment which was dependant upon the number of effector cells treated and on the MMC dosage used. MMC induced bystander killing was cell type specific with no bystander effect occurring in cervical cancer cells, although the drug did induce direct cytotoxicity in these cells per se.

Chemotherapeutic drugs induce expression of death receptors [21,27,28], and their role in drug induced bystander effect is still not clear. MMC treatment increased Fas expression in HepG2 cells and these cells were sensitive to Fas/FasL mediated apoptosis [29,30]. Whereas, Hep3B cells are reported to be resistant to Fas mediated apoptosis [21,31]. Our data clearly demonstrated that Hep3B cells express Fas, though reduced percentage of cells displayed the expression and this observation was in agreement with the report of Lamboley et al [31]. Moreover, in MMC treated Hep3B cells, no significant increase in the levels of Fas was detected. Therefore, it is unlikely that the Fas-FasL pathway was involved in MMC induced bystander effect in co-cultures of effector Hep3B and target Hep3B cells. Concurrently, MMC treatment caused a significant increase in TRAIL receptor DR4 in Hep3B cells. In the presence of neutralizing anti-TRAIL antibody, the MMC induced bystander killing of Hep3B diminished, indicating involvement of the TRAIL pathway in mediating a bystander effect. Interestingly, the presence of neutralizing anti-FasL antibody prevented bystander killing in HepG2 cells, but cell death was not prevented in the presence of neutralizing anti-TRAIL antibody. In mixed co-cultures of effector Hep3B cells and target HepG2-EGFP cells, there was also a decrease in bystander killing in the presence of neutralizing anti-FasL antibody, suggesting involvement of the Fas-FasL pathway. Additionally, the significant increase in FasL and TRAIL in CM of MMC treated effector cells was suggestive of involvement of secreted form of these ligands being responsible for transferring a bystander signal through the medium. The involvement of these ligands was reconfirmed by culturing MMC treated effector cells with/without neutralizing anti-FasL and/or anti-TRAIL antibody in target HepG2 and Hep3B cells, respectively (Additional file 4). The pro-apoptotic role of secreted forms of FasL and TRAIL in hepatocellular carcinoma has been reported [32-34], though there are contradictory reports suggesting an anti-apoptotic role of secreted FasL [35-37]. It has been reported that the cleaved product of human FasL has cytotoxic activity [32], but it is relatively weaker than the membrane associated FasL [38]. Also, the cytotoxic activity is enhanced if the death ligands are present in cross linked or aggregated form [32-35]. Thus, the detection of the high molecular weight form of FasL (≈ 48 kDa) in CM from treated HepG2 cells suggested its involvement in bystander killing (Additional file 1). The significance of secreted death ligands in bystander effect was further confirmed by treatment with EDTA. As shown in Fig. 8A, in the presence of EDTA, the bystander killing decreased in medium transfer, as well as under co-culturing conditions (Fig. 8B). Treatment with EDTA led to a decreased abundance of FasL in CM that correlated well with diminished PARP cleavage in HepG2 cells co-cultures (Fig. 8C and Fig. 8D) Although, HepG2 effector cells also released TRAIL, the amount was insignificant in comparison to the amount of FasL released in the medium.

For a chemotherapeutic drug to be effective in eliminating tumor completely, the drug or its effect should reach not only the cells in periphery, but also the inner cellular mass of the tumor. One of the possibilities to achieve this was to explore the feasibility of diffusion of released cytotoxic effector molecules from the tumor cells exposed to the drug directly. Therefore, to verify that indeed released cytotoxic effector molecules from the treated cells were able to diffuse through semisolid medium thereby mimicking *in-vivo *conditions, we performed soft-agarose colony formation assays. Because MMC treated effector HepG2 secret FasL, we hypothesized that this ligand should diffuse through semisolid medium. Interestingly, we found that in the plate in which treated HepG2 effector cells were added in the centre well, colony size was smaller and they were of distinct shapes, suggestive of diminished cell growth (Fig. 11B). The decrease in size and alterations in shape of colonies was prevented when the treated effector cells were added in the centre well along with neutralizing anti-FasL antibody (Additional file 2d).

## Conclusion

**Figure 12:**
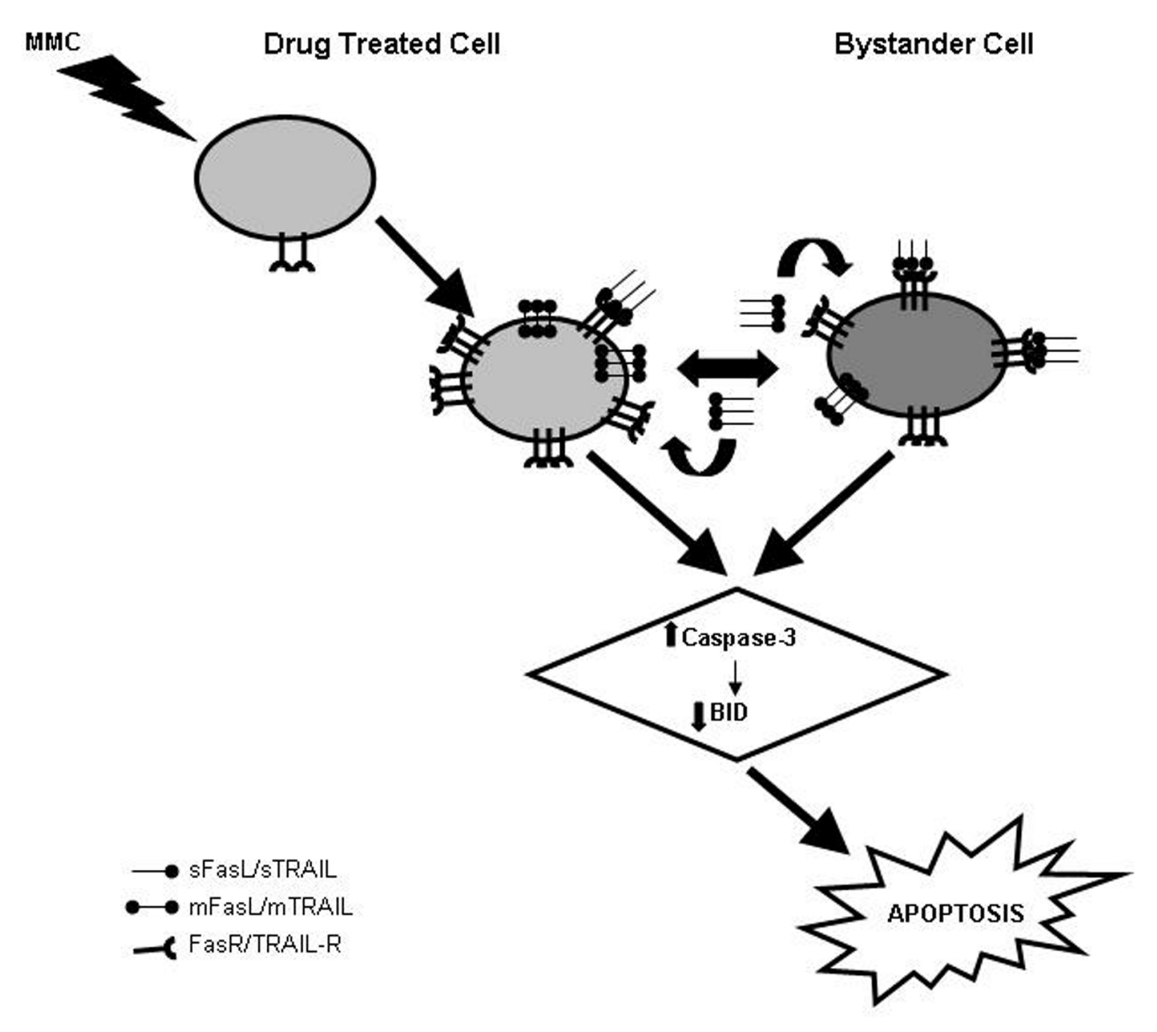
**Proposed model**. The generation of MMC induced bystander signals deciphering function of membrane bound as well as secreted form of death ligands.

Our results support the idea that bystander killing in hepatoma cells is mediated by membrane bound as well as secreted form of death ligands. This is further assisted by increased expression of corresponding death receptors. Moreover, since FasL upregulates Fas [39,40], we propose that the release of the secreted form of the death ligand from effector cells is able to boost the expression of death receptors on bystander cells (Fig. 12). The work described in this study suggests that MMC induces direct cytotoxicity in cervical cancer and hepatoma cells. However, amplification of killing is not achieved in cervical cancer cells because death signals are not propagated by bystander effect. Therefore, MMC may potentially be of more therapeutic value in treating hepatocellular carcinoma than cervical cancers because of its ability to initiate bystander effect. Generally, at early stages of tumor development the cell population is likely to be homogeneous, whereas in advanced stages it is likely to be heterogeneous. Since MMC is able to induce bystander killing in both homogeneous and heterogeneous cellular models, it is likely to be more effective in treatment forearly and advanced staged HCCs.

## Abbreviations

HCC: Hepatocellular carcinoma; MMC: Mitomycin C; MTT: Methyle Thiazole Tetrazolium; CM: Conditioned Medium; Mab: Monoclonal antibody; EDTA: Ethylene Diaminetetra acetic acid.

## Competing interests

The authors declare that they have no competing interests.

## Authors' contributions

RK performed most of the experiments and prepared the manuscript. AS and AKA participated in performing few experiments and helped to draft the manuscript. MKB conceived the study, participated in its design and coordination, corrected the manuscript and supervised the project. All authors read and approved the final manuscript.

## Supplementary Material

Additional file 1 **Secreted form of FasL detected in CM obtained from MMC treated HepG2 cells**. Immunoblot of CM obtained from HepG2 cells after MMC treatment and EDTA pre-treatment followed by MMC treatment. Blots were probed with anti FasL antibody. Higher molecular weight form of FasL was detected. Click here for file

Additional file 2 **Secreted FasL from effector HepG2 cells is able to inhibit the growth of target HepG2 cell colonies in soft agarose colony formation assay**. Panel (a) untreated effector HepG2 cells were added in the center well, panel (b) treated effector HepG2 cells were added in the center well, panel (c) bioactive recombinant FasL (6 μg) was added in the center well and (d) MMC treated effector HepG2 cells along with neutralizing anti FasL antibody (4 μg/ml) were added in the center well Click here for file

Additional file 3 **Colony formation assay by using medium transfer strategy**. Colonies of target HepG2 cells after medium from treated and/or untreated effector HepG2 cells was added to the target cells. Briefly, effector HepG2 cells (7 × 10^5^) were seeded in a 60 mm tissue culture dish. The cells were treated with MMC (150 nM) for 24 h. Then medium containing MMC was decanted, cells were washed with medium and fresh medium without drug was added to the cells. Cells were then cultured for additional 48 h and subsequently this medium was used to culture the target cells. Target cells (0.25 × 10^5^) were seeded in a 35 mm tissue culture dish, allowed to adhere at 37°C for 24 h before medium from untreated and/or treated effector HepG2 cells with/without neutralizing anti-FasL antibody was added Panel (a) medium from untreated HepG2 cells was added, panel (b) medium from MMC treated HepG2 cells was added and panel (c) medium from MMC treated HepG2 cells supplemented with neutralizing anti-FasL antibody (2 μg/ml) was added. Cells were allowed to grow for 12 days. Medium was changed once in 6 days. After 12 days medium was decanted, colonies were fixed with 3.7% paraformaldehyde and stained with 0.005% crystal violet stain. Click here for file

Additional file 4 **MMC induced apoptosis of HepG2 and Hep3B cell is mediated by FasL and TRAIL respectively**. Photomicrographs of effector HepG2 and Hep3B cells. Panel (a) untreated cells, panel (b) cells treated with MMC (150 nM) and panel (c) cells treated with MMC (150 nM) and then grown in medium containing neutralizing anti-FasL antibody in case of HepG2 cells and anti-TRAIL antibody in case of Hep3B cells. Click here for file
