# Supplementary figures and images for: Mitomycin C induces bystander killing in homogeneous and heterogeneous hepatoma cellular models
Source: Mol Cancer. 2009 Oct 21;8:87. doi: 10.1186/1476-4598-8-87 (PMC2770032; doi:10.1186/1476-4598-8-87)

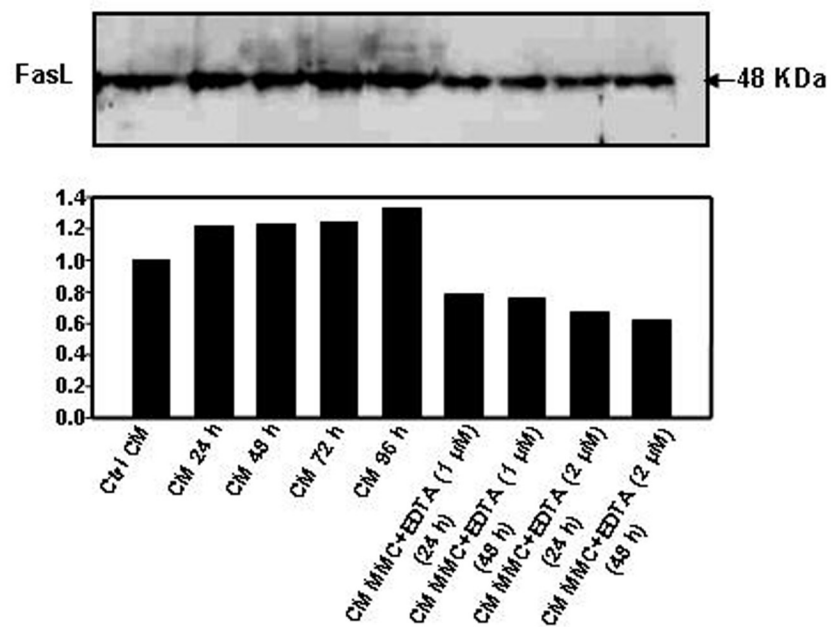

Supplement: Additional file 1 — Secreted form of FasL detected in CM obtained from MMC treated HepG2 cells. Immunoblot of CM obtained from HepG2 cells after MMC treatment and EDTA pre-treatment followed by MMC treatment. Blots were probed with anti FasL antibody. Higher molecular weight form of FasL was detected. [file 1476-4598-8-87-S1.PDF]

**a**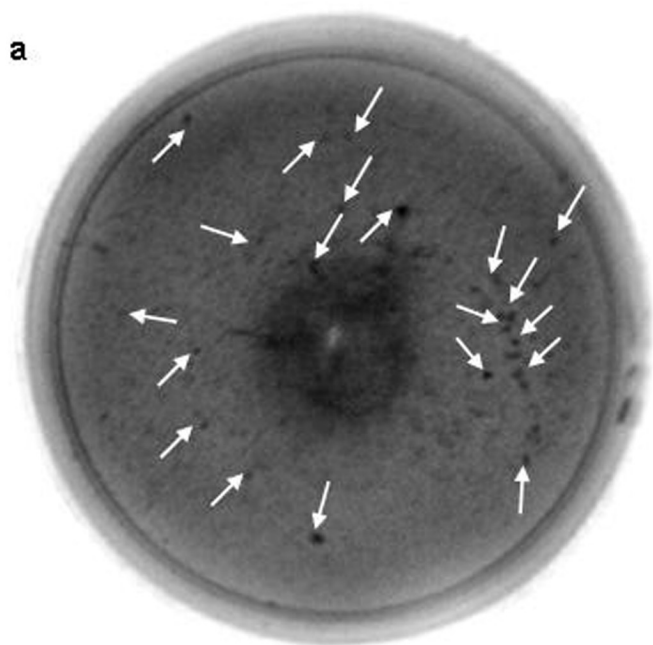**b**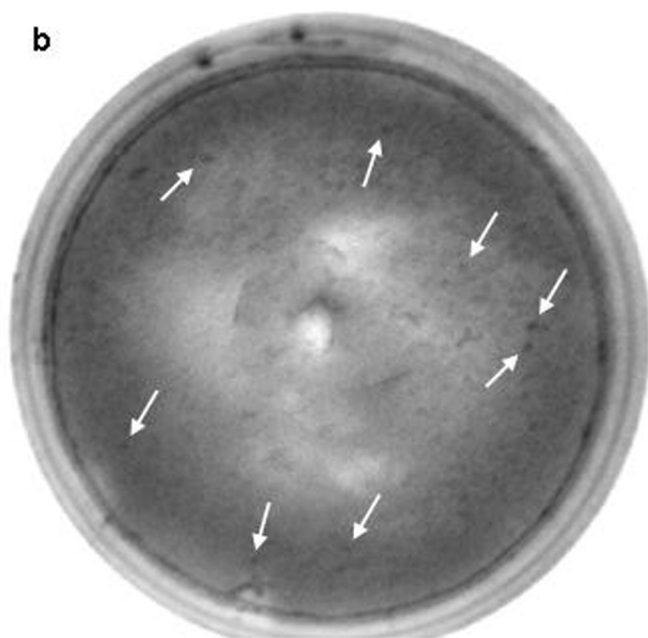**c**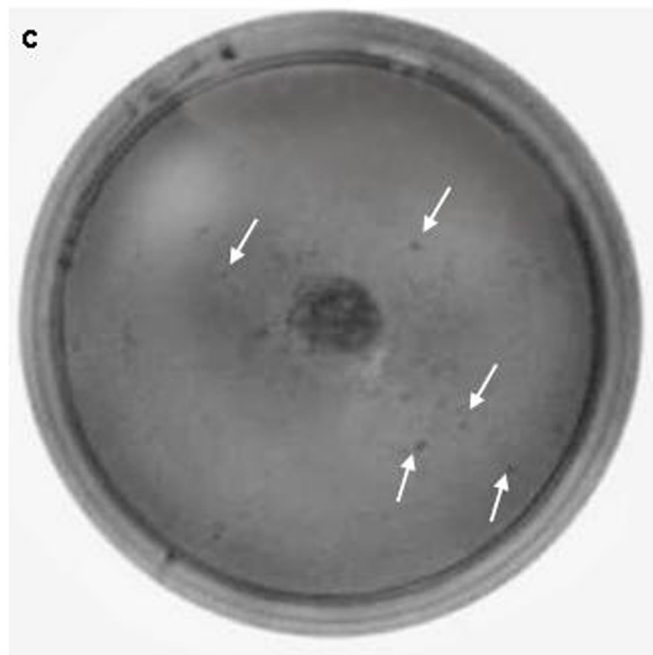**d**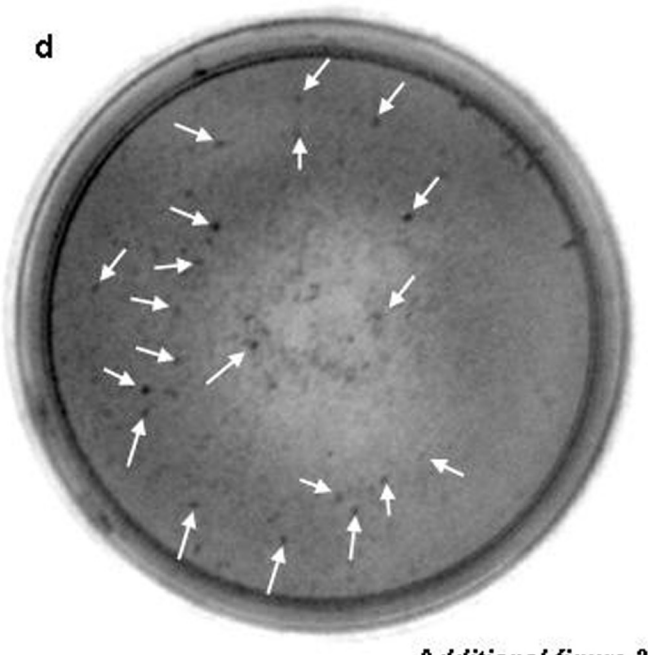

Supplement: Additional file 2 — Secreted FasL from effector HepG2 cells is able to inhibit the growth of target HepG2 cell colonies in soft agarose colony formation assay. Panel (a) untreated effector HepG2 cells were added in the center well, panel (b) treated effector HepG2 cells were added in the center well, panel (c) bioactive recombinant FasL (6 μg) was added in the center well and (d) MMC treated effector HepG2 cells along with neutralizing anti FasL antibody (4 μg/ml) were added in the center well [file 1476-4598-8-87-S2.PDF]

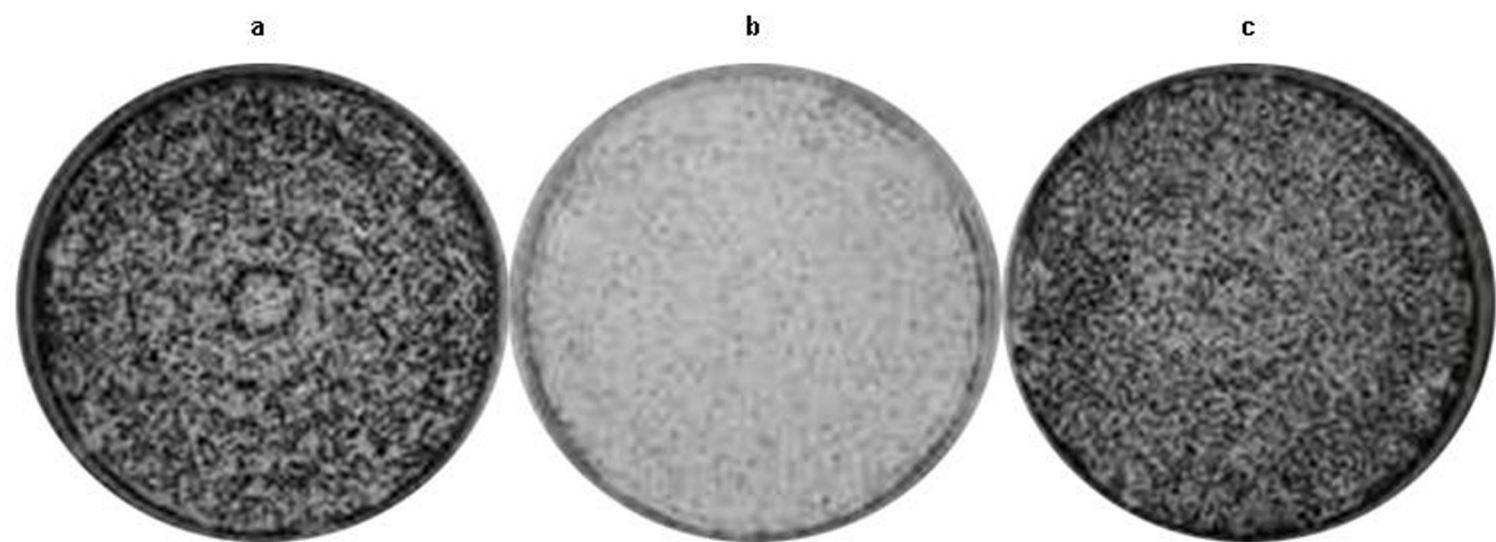

Supplement: Additional file 3 — Colony formation assay by using medium transfer strategy. Colonies of target HepG2 cells after medium from treated and/or untreated effector HepG2 cells was added to the target cells. Briefly, effector HepG2 cells (7 × 10^5^) were seeded in a 60 mm tissue culture dish. The cells were treated with MMC (150 nM) for 24 h. Then medium containing MMC was decanted, cells were washed with medium and fresh medium without drug was added to the cells. Cells were then cultured for additional 48 h and subsequently this medium was used to culture the target cells. Target cells (0.25 × 10^5^) were seeded in a 35 mm tissue culture dish, allowed to adhere at 37°C for 24 h before medium from untreated and/or treated effector HepG2 cells with/without neutralizing anti-FasL antibody was added Panel (a) medium from untreated HepG2 cells was added, panel (b) medium from MMC treated HepG2 cells was added and panel (c) medium from MMC treated HepG2 cells supplemented with neutralizing anti-FasL antibody (2 μg/ml) was added. Cells were allowed to grow for 12 days. Medium was changed once in 6 days. After 12 days medium was decanted, colonies were fixed with 3.7% paraformaldehyde and stained with 0.005% crystal violet stain. [file 1476-4598-8-87-S3.PDF]

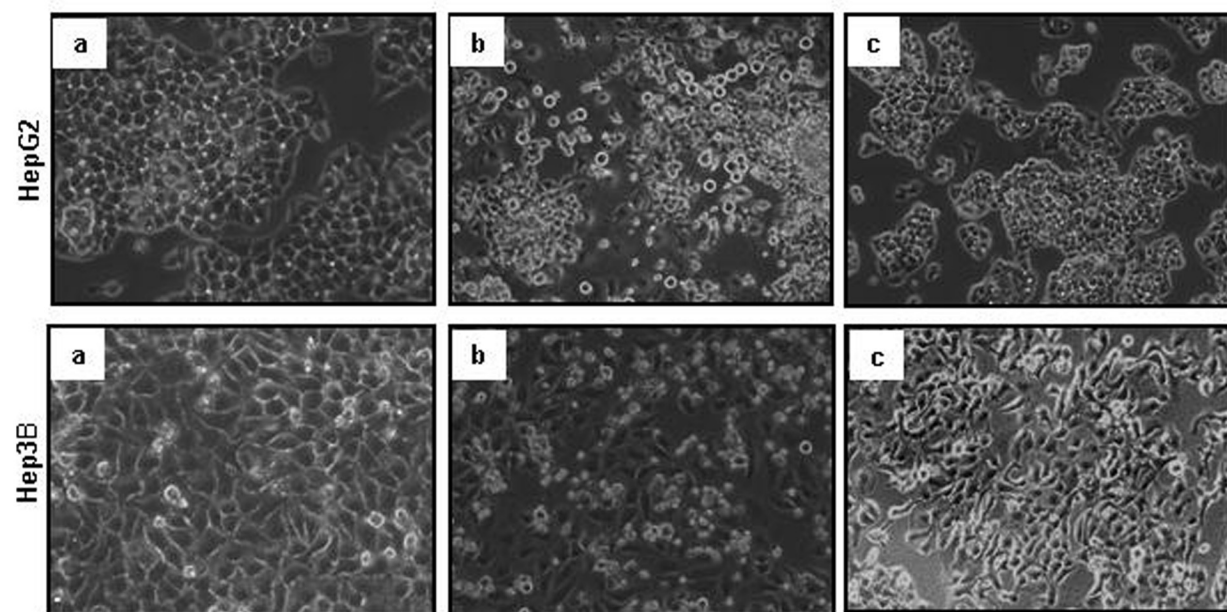

Supplement: Additional file 4 — MMC induced apoptosis of HepG2 and Hep3B cell is mediated by FasL and TRAIL respectively. Photomicrographs of effector HepG2 and Hep3B cells. Panel (a) untreated cells, panel (b) cells treated with MMC (150 nM) and panel (c) cells treated with MMC (150 nM) and then grown in medium containing neutralizing anti-FasL antibody in case of HepG2 cells and anti-TRAIL antibody in case of Hep3B cells. [file 1476-4598-8-87-S4.PDF]
